# Subacute Oral Administration of *Clinacanthus nutans* Ethanolic Leaf Extract Induced Liver and Kidney Toxicities in ICR Mice

**DOI:** 10.3390/molecules25112631

**Published:** 2020-06-05

**Authors:** Abdullahi Aliyu, Mohd Rosly Shaari, Nurul Syahirah Ahmad Sayuti, Mohd Farhan Hanif Reduan, Shanmugavelu Sithambaram, Mustapha Mohamed Noordin, Khozirah Shaari, Hazilawati Hamzah

**Affiliations:** 1 Department of Veterinary Pathology and Microbiology, Faculty of Veterinary Medicine, Universiti Putra Malaysia, Serdang 43400, Selangor, Malaysia; nurulsyahirah0806@gmail.com (N.S.A.S.); farhan.h@umk.edu.my (M.F.H.R.); noordinmm@upm.edu.my (M.M.N.); 2 Department of Veterinary Pathology, Faculty of Veterinary Medicine, City Campus Complex, Usmanu Danfodiyo University, Sokoto 840212, Sokoto State, Nigeria; 3 Animal Science Research Centre, Malaysian Agricultural Research and Development Institute Headquarter, Serdang 43400, Selangor, Malaysia; rosly@mardi.gov.my (M.R.S.); shan.sithambaram@gmail.com (S.S.); 4 Department of Chemistry, Faculty of Science and Environmental Studies, Universiti Putra Malaysia, Serdang 43400, Selangor, Malaysia; khozirah@upm.edu.my; 5 Laboratory of Natural Products, Institute of Bioscience, Universiti Putra Malaysia, Serdang 43400, Selangor, Malaysia

**Keywords:** *Clinacanthus nutans*, myricetin, isookanin, orientin, vitexin, ICR mice, acute toxicity, subacute toxicity, histopathology, biochemical parameters

## Abstract

This study investigated the leaves of *Clinacanthus nutans* for its bioactive compounds and acute and subacute toxicity effects of *C. nutans* ethanolic leaf extract (CELE) on blood, liver and kidneys of ICR mice. A total of 10 8-week-old female mice were divided into groups A (control) and B (2000 mg/kg) for the acute toxicity study. A single dose of 2000 mg/kg was administered to group B through oral gavage and mice were monitored for 14 days. In the subacute toxicity study, mice were divided into five groups: A (control), B (125 mg/kg), C (250 mg/kg), D (500 mg/kg) and E (1000 mg/kg). The extract was administered daily for 28 days via oral gavage. The mice were sacrificed, and samples were collected for analyses. Myricetin, orientin, isoorientin, vitexin, isovitexin, isookanin, apigenin and ferulic acid were identified in the extract. Twenty-eight days of continuous oral administration revealed significant increases (*p* < 0.05) in creatinine, ALT and moderate hepatic and renal necrosis in groups D and E. The study concluded that the lethal dose (LD_50_) of CELE in mice is greater than 2000 mg/kg and that repeated oral administrations of CELE for 28 days induced hepatic and renal toxicities at 1000 mg/kg in female ICR mice.

## 1. Introduction

The use of plant-based products in both traditional and modern societies as herbal remedies or crude drugs, or as purified compounds have a long history [1]. Phytoresearches provide significant classes of drugs that are used in the treatment of various illnesses in both humans and animals including cancers, malaria, HIV/AIDS and diabetes. In the recent past, medicinal plants, such as *C. nutans*, are gaining increasing attention in both traditional and modern societies [1]. Most of these plants are widely distributed and consumed globally, particularly in developing countries, where larger proportion depend on plants and their products for their primary health care challenges [2] and chronic diseases. This is perhaps because of poverty, the increasing cost of modern medicines and little awareness of the plants’ side effects [3].

*Clinacanthus nutans* is a known herbal plant that belongs to the family Acanthaceae, which consists of 250 genera and about 2500 species, which are mostly tropical herbs or shrubs, and epiphytes in some cases [2]. The genus Clinacanthus consists of two species, *C. nutans* (Burm. F.) Lindau and *C. siamensis* Bremek, both of which are found throughout Southeast Asia. The two species have different pharmacological characteristics, molecular aspect and anti-herpes simplex virus (HSV) types 1 and 2 activities [4]. *C. nutans* is locally known as “Sabah snake grass” or “rumput belalai gajah” (in Malaysia), “Phayo yo” (in Thailand) and “Dandang Gendis” (in Indonesia) [5]. The plant has been initially used to treat poisonous snake bites; however, it was later identified to possess potential antiviral properties [6]. It is traditionally used in Thailand for the treatment of various illnesses including cancers, skin rashes, snake and insects bites, diabetes mellitus, diarrhoea, as an anti-viral agent against HSV and varicella-zoster virus (VZV) [2,7]. The mechanism of action of this plant is attributed to its anti-cell lysis property rather than an antineuromuscular transmission blocker [6]. The wide range of pharmacological activities associated with *C. nutans* could be attributed to the bioactive compounds reported in different parts of the plant, including saponins, glycosides, steroids and flavonoids [8,9]. Recently, Hao et al. [10] reported that preparations of silver and gold nanoparticles using aqueous leaf extract of *C. nutans* Lindau demonstrated higher analgesic and muscle relaxant activities at concentrations of 50, 100 and 150 mg/kg body weight in BALB/c mice, compared to the methanolic extract of the plant at concentrations of 100, 200 and 400 mg/kg [10].

Toxicity evaluation of *C. nutans* therefore becomes very necessary in order to ascertain its safety levels that should be used for the treatment of various illnesses. The current literature mostly reported the toxicity effects of the aqueous leaf extracts of the plants [11,12], while a few others that employed the methanolic/ethanolic leaf extracts reported that the plant is not associated with toxicity effects mostly based on the evaluation of body weight changes, haematological and serum biochemical parameters [9,13,14], while the effects of the extracts on the histology of liver and kidneys, which were the primary organs involved in the detoxification and excretion of potential toxic substances from the body, were not reported.

Murugesu at al. [15] investigated the in vitro toxic effect and the lethal concentration of non-polar fraction of *C. nutans* at concentrations of 15.63, 31.25, 62.5, 125, 250 and 500 µg/mL on zebrafish embryos at 72 hpf. The results showed that the median lethal concentration (LC_50_) was found to be 75.49, which was harmful [15] according to the organization for economic co-operation and development (OECD) guideline. The concentration of 500 µg/mL was toxic to the embryos showing 100% mortality after 24 h. Other malformations reported by Murugesu et al. [15] due to hexane extract of *C. nutans* in zebrafish embryos included spinal curvature, oedema, malformed yolk sac, as well as reduced hatchability and growth retardation [15]. These in vitro toxic lesions make it necessary to study further the in vivo toxicity effects of *C. nutans* extract in laboratory animals.

Currently, there is paucity of information on the bioactive compounds, nutritional composition and toxicity effects of *C. nutans* cultivated in Pahang-Malaysia. Some of the previous studies [3,9] reported the groups of phytochemical compounds (e.g., saponins, glycosides, steroids, flavonoids, etc.) in *C. nutans*. However, the specific phytocompounds in this plant cultivated in Pahang-Malaysia have not been adequately investigated. Besides, there is no uniformity in the previous reports that investigated the phytochemical compounds of the plant [16,17,18], possibly due to the differences in agro-climatic conditions, genetic factors, differences in cultivation techniques, drying and extraction methods employed [19,20]. Förster et al. [21] reported that the secondary metabolites of plants are highly susceptible to various environmental conditions, including temperature regulation, water quality and cycling, carbon and nutrient cycle, etc. This makes it necessary to verify the phytochemical compounds of medicinal plants in each field trial in the various cultivation areas [21].

Consequently, this study investigated the bioactive compounds of *C. nutans* ethanolic leaf extract cultivated and collected from Pahang-Malaysia using a liquid chromatography-electrospray ion mass spectrometer (LCESI-MS/MS). Moreover, the detailed effects of single as well as repeated oral administration of CELE on the blood parameters as well as histology of liver and kidney of female institute of cancer research (ICR) mice were investigated in this study. This study would provide reliable data on the bioactive compounds of *C. nutans* cultivated in Pahang-Malaysia, at the same time provide details possible toxic effects of CELE on the histology of liver and kidneys of female ICR mice, which could be useful as a guide in selecting appropriate doses for future treatment studies using the plant extract.

## 2. Results

### 2.1. Liquid Chromatography-Mass Spectrometry (LC-MS)

**Figure 1:**
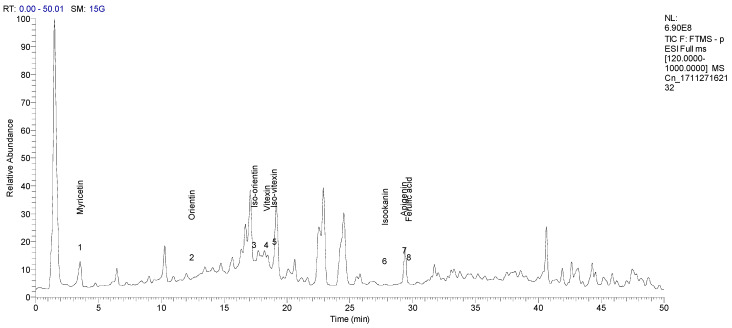
Total ion chromatograms (TIC) of the compounds in *Clinacanthus nutans* ethanolic leaf extract (CELE).

**Figure 2:**
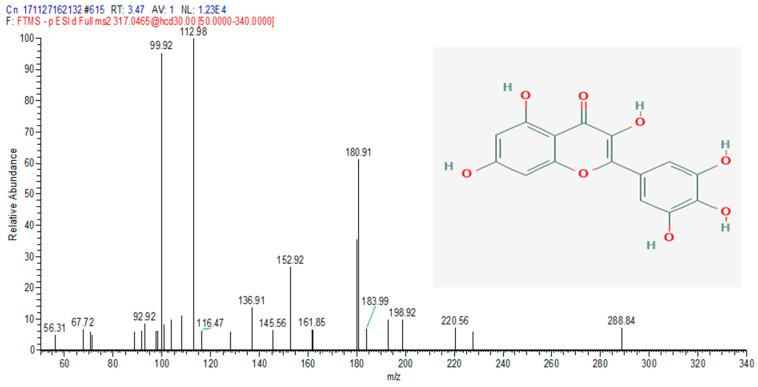
Myricetin.

**Figure 3:**
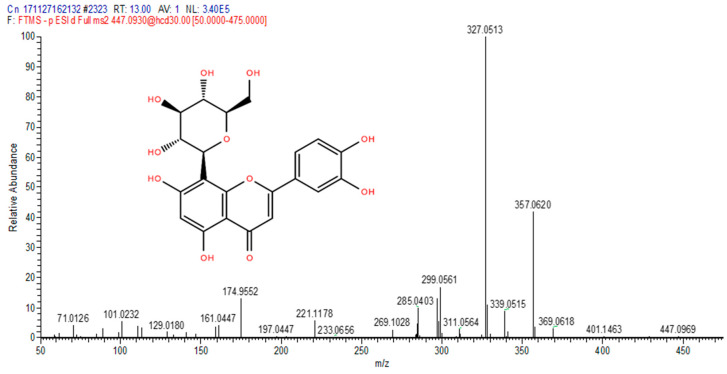
Orientin.

**Figure 4:**
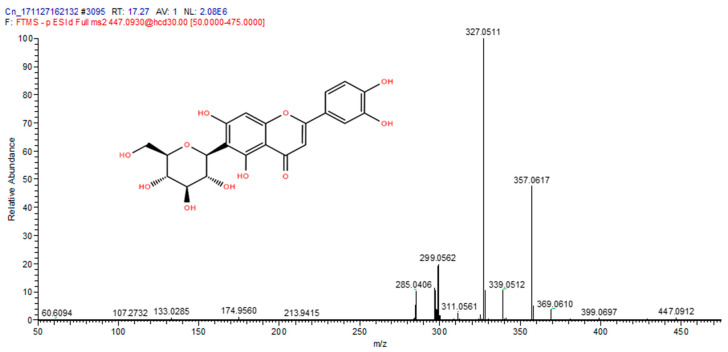
Isoorientin.

**Figure 5:**
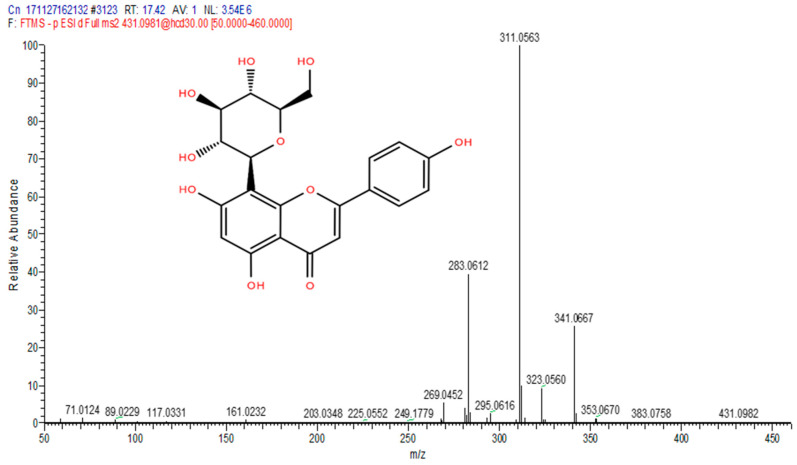
Vitexin.

**Figure 6:**
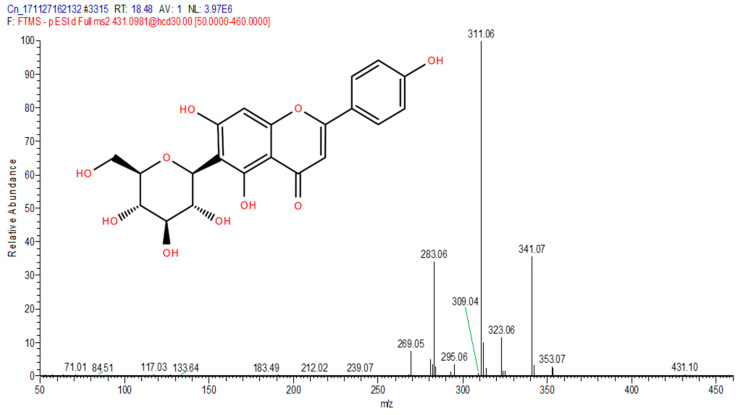
Isovitexin.

**Figure 7:**
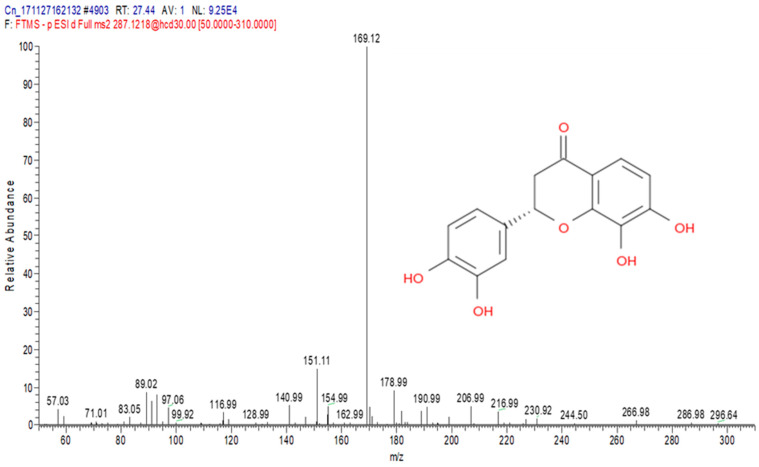
Isookanin.

**Figure 8:**
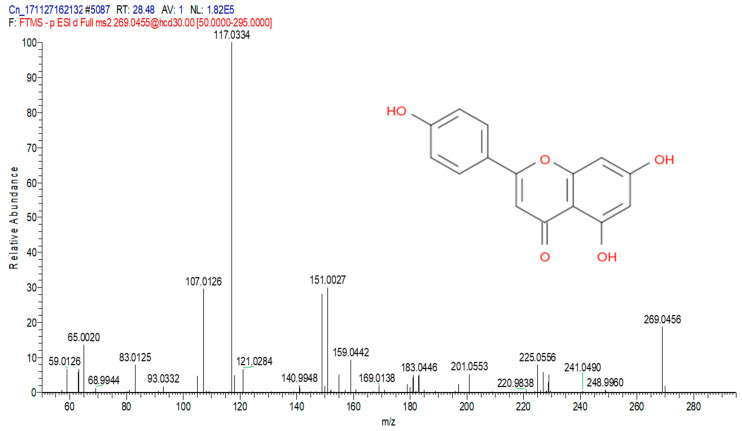
Apigenin.

**Figure 9:**
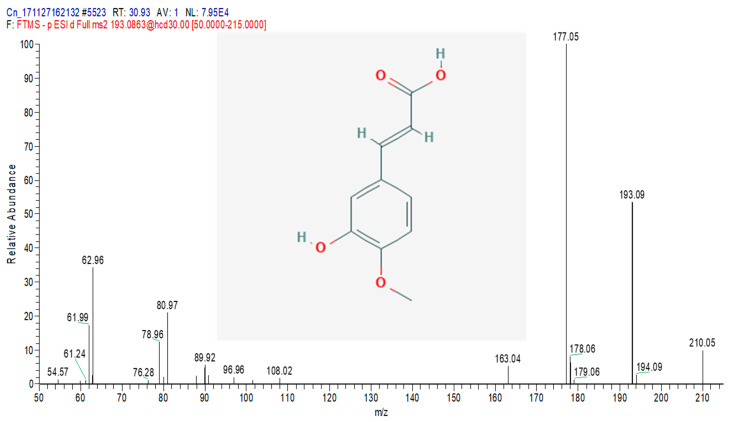
Ferulic acid.

The total ion chromatograms (TIC) of the compounds identified in the sample of *Clinacanthus nutans* ethanolic leaf extract (CELE) are demonstrated in Figure 1. The identities of eight compounds were determined along with their retention time, protonated molecular ions and characteristic fragment ions (Table 1). The compounds identified based on the LC-MS and MS/MS data analysis include myricetin, orientin, isoorientin, vitexin, isovitexin, isookanin, apigenin and ferulic acid (Table 1). The total ion chromatogram and the structure of the eight compounds identified are presented below, in Figure 1, Figure 2, Figure 3, Figure 4, Figure 5, Figure 6, Figure 7, Figure 8 and Figure 9. The compounds were identified by comparing their masses (*m*/*z*), fragment ions and mass spectra with those reported in the mass bank (metabolite database).

### 2.2. Acute Toxicity Study

#### 2.2.1. Body Weight Gain

**Figure 10:**
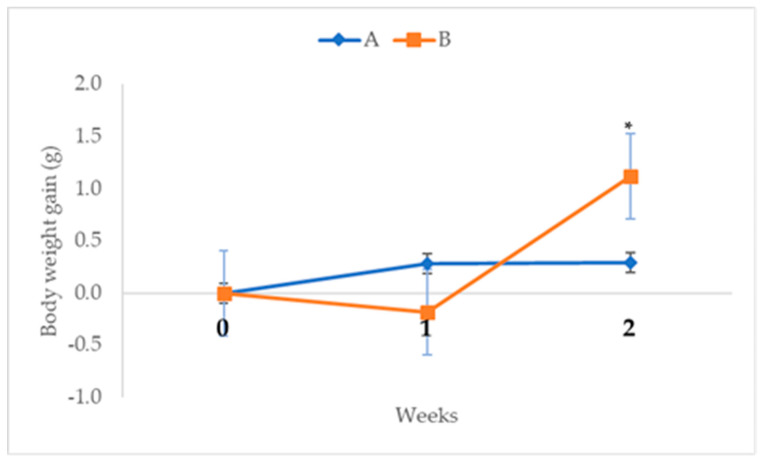
Weekly body weight gain (g) of female ICR mice in acute toxicity study of CELE. Key: CELE = *Clinacanthus nutans* ethanolic leaf extract, A = control, B = 2000 mg/kg CELE, * = significantly different at *p* < 0.05.

The effects of oral administration of 2000 mg/kg CELE on the mean body weight gain of mice is presented on Figure 10. Repeated measures ANOVA with a Greenhouse-Geisser correction and Bonferroni post hoc test showed significant (*p* < 0.05) differences in the body weight gain of the treated mice across the 14 days experimental period. There was 267% decrease (*p* > 0.05) as well as 189% increase (*p* < 0.05) in the body weight gain of the mice in group B at weeks 1 (−0.47 ± 0.60 g) and 2 (0.83 ± 0.64 g) of the experiment, respectively, compared to group A at weeks 1 (0.28 ± 0.60 g) and 2 (0.29 ± 0.21 g) (Figure 10).

#### 2.2.2. Relative Organs Weight

The effects of oral administration of 2000 mg/kg CELE on the relative organ weight of female ICR mice are presented on Table 2. There were no statistically significant (*p* > 0.05) differences between groups in the relative organs weights of the mice treated with CELE throughout the 14 days, as determined by Student’s *t* test (Table 2). The organ to body weight ratios of liver (5.70 ± 0.33) was 6.3%, which was higher (*p* > 0.05) in group B (5.70 ± 0.33) compared to A (5.39 ± 0.44), while the organ to body weight ratios of right kidney (0.60 ± 0.04), spleen (0.57 ± 0.05), heart (0.62 ± 0.10), and lungs (1.07 ± 0.14) were 15.49%, 3.39%, 4.62%, and 28.19%, which were lower (*p* > 0.05) in group B compared to the relative organs weights of right kidney (0.71 ± 0.08), spleen (0.59 ± 0.10), heart (0.65 ± 0.07) and lungs (1.49 ± 0.17) in group A. (Table 2).

#### 2.2.3. Haematological Parameters

The effects of oral administration of 2000 mg/kg CELE on the haematological parameters of female ICR mice is presented on Table 3. There were no statistically significant (*p* > 0.05) differences between groups in the haematological parameters of the mice treated with 2000 mg/kg CELE throughout the 14-day observation period, as determined by Student’s *t* test (Table 3). However, the haemoglobin concentration was 2.8% higher (*p* > 0.05) in group B (181.80 ± 5.70 g/L) compared to A (176.80 ± 2.87 g/L) and the values of the platelets were 4.4% lower (*p* > 0.05) in group B (1252.00 ± 204.80 × 10^9^/L) compared to A (1308.40 ± 32.66 × 10^9^/L) (see Table 3). Moreover, the values for the total white blood cell counts (TWBC), neutrophils, lymphocytes and monocytes between groups A and B were comparable (*p* > 0.05) (see Table 3).

#### 2.2.4. Biochemical Parameters

The effects of oral administration of 2000 mg/kg CELE on the plasma biochemical parameters of ICR mice is presented on Table 4. The plasma level of creatinine was 17.04% lower (*p* > 0.05) in group B (29.20 ± 1.50 µmol/L) compared to A (35.20 ± 2.42 µmol/L). Furthermore, there was 185% significant (*p* < 0.05) increase in alanine aminotransferase (ALT) in group B (289.60 ± 30.99 U/L) compared to A (101.60 ± 5.75 U/L), a 101.5% significant (*p* < 0.05) increase in aspartate aminotransferase (AST) in group B (344.60 ± 45.92 U/L) compared to A (171.00 ± 10.89 U/L), as well as a 67.6% significant (*p* < 0.05) increase in creatinine kinase (CK) in group B (549.20 ± 66.05 U/L) compared to A (327.60 ± 35.71 U/L). The plasma level of total proteins was 5.82% lower (*p* > 0.05) in group B (59.86 ± 2.17 g/L) compared to A (63.56 ± 1.71 g/L). Moreover, the plasma albumin level was 6.2% lower (*p* > 0.05) in group B (32.00 ± 0.45 g/L) compared to A (34.10 ± 0.73 g/L) (see Table 4).

#### 2.2.5. Liver Lesion Scoring

The effects of oral administration of 2000 mg/kg CELE on the histology of liver of female ICR mice are presented on Table 5. Mann-Whitney U test revealed no statistically significant (*p* > 0.05) differences in the liver lesion score between the control and treatment groups (Table 5). 

#### 2.2.6. Kidney Lesion Scoring

The effects of oral administration of 2000 mg/kg CELE on the histology of kidneys of female ICR mice are presented on Table 6. There were no statistically significant (*p* > 0.05) differences in the kidney lesion score between the control and treatment groups as shown by Mann-Whitney U test (Table 6). 

### 2.3. Subacute Toxicity Study

#### 2.3.1. Body Weight Gain

**Figure 11:**
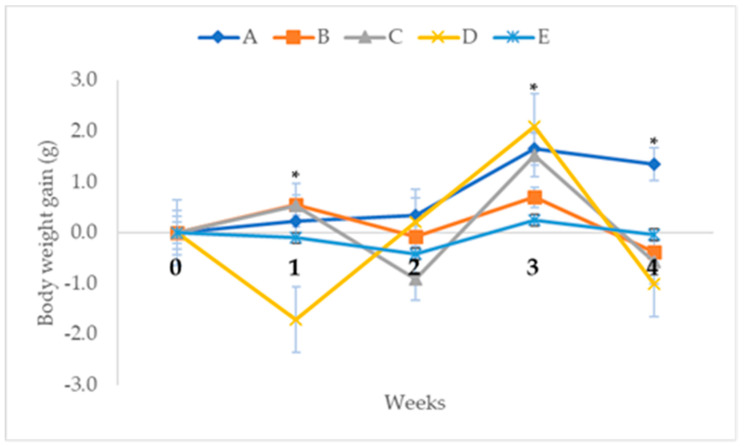
Average (mean ± SEM) weekly body weight gain (g) of female ICR mice in subacute toxicity study of CELE. Key: A = control, B = 125 mg/kg CELE, C = 250 mg/kg CELE, D = 500 mg/kg CELE, E = 1000 mg/kg CELE, significantly different at *p* < 0.05, SEM = standard error of mean, CELE = *Clinacanthus nutans* ethanolic leaf extract.

The effects of repeated oral administration of different doses of CELE daily for 28 days on the average body weight gain of female ICR mice are presented on Figure 11. Repeated measures ANOVA with a Greenhouse-Geisser correction and Bonferroni post hoc test showed significant (*p* < 0.05) differences in the body weight gain of the treated mice across the 4-week experimental period (Figure 11). There was 136.21% increased (*p* > 0.05) weight gain in group B at week 1 (0.55 ± 0.98 g), as well as 121.14%, 57.78% and 127.81% reductions in the body weight gains of the mice in group B at weeks 2 (−0.07 ± 0.43; *p* > 0.05), 3 (0.70 ± 0.61; *p* < 0.05) and 4 (−0.38 ± 0.34; *p* < 0.05) of the experiment, respectively, compared to group A at weeks 1 (0.23 ± 0.36 g), 2 (0.35 ± 0.31 g), 3 (1.66 ± 0.24 g) and 4 (1.35 ± 0.54 g) (Figure 11). Furthermore, there was 133.62% increase in the body weight gain in group C, at week 1 (0.54 ± 0.83 g; *p* > 0.05) of the experiment, as well as 360% and 142.16% reductions in the average body weight gains in group C at weeks 2 (−0.91 ± 0.85 g; *p* < 0.05) and 4 (−0.57 ± 0.26 g; *p* < 0.05) of the experiment respectively, compared to group A at weeks 1, 2 and 4 (see Figure 11). Similarly, the average body weight gain of the mice in group D were 838.79%, 40.57% and 175% lower at weeks 1 (−1.71 ± 1.54 g; *p* < 0.05), 2 (0.21 ± 0.75 g; *p* > 0.05) and 4 (−1.01 ± 1.29 g; *p* < 0.05) of the experiment, respectively, compared to group A at weeks 1, 2 and 4 (see Figure 11). Correspondingly, there were 142.24%, 220%, 85.16% and 102.96% reductions in the body weight gains of the mice in group E at weeks 1 (−0.10 ± 0.45 g; *p* > 0.05), 2 (−0.42 ± 0.66 g; *p* > 0.05), 3 (0.25 ± 0.40 g; *p* < 0.05) and 4 (−0.04 ± 0.28 g; *p* < 0.05) of the experiment, respectively, compared to group A at weeks 1, 2, 3 and 4 (see Figure 11).

#### 2.3.2. Relative Organs Weight

The effects of repeated oral administration of different doses of CELE daily for 28 days on the relative organs weight of female ICR mice are presented on Table 7. There were no statistically significant (*p* > 0.05) differences between groups in the relative organ weights of the mice treated with CELE daily for 28 days, as determined by one-way ANOVA, except for the uterus (Table 7). However, the relative weights of liver were 18.5%, 18.8% and 14% lower (*p* > 0.05; F = 0.964) in groups B (5.55 ± 0.38), C (5.53 ± 0.34) and E (5.86 ± 0.41), respectively, compared to A (6.81 ± 0.85). The relative weights of kidney were 2.5% and 8.9% higher (*p* > 0.05; F = 0.651) in groups D (0.81 ± 0.05) and E (0.86 ± 0.13), respectively, compared to A (0.79 ± 0.11) (see Table 7). The relative organ weight of uterus were 16.6%, 37.9%, 39.1% and 15.3% lower (*p* < 0.05; F = 1.487) in groups B (1.96 ± 0.26), C (1.46 ± 0.29), D (1.43 ± 0.15) and E (1.99 ± 0.37), respectively, compared to group A (2.35 ± 0.25) (see Table 7). 

#### 2.3.3. Haematological Parameters

The effects of repeated oral administration of different doses of CELE daily for 28 days on the haematological parameters of female ICR mice is presented on Table 8. There were statistically significant (*p* < 0.05) differences in the haematological parameters between groups as determined by one-way ANOVA. The Tukey post hoc test showed that the mice treated with CELE had 3% and 9.3% significant (*p* < 0.05) increases in the values of MCV and MCH, respectively, in group B (68.25 ± 1.80 fl; 17.33 ± 0.56 pg) compared to A (65.80 ± 0.97 fl; 15.86 ± 0.28 pg). There were also 5.5% and 7.3% significant decreases (*p* < 0.05) in the values of MCV in groups D (62.20 ± 1.69 fl) and E (61.00 ± 1.48 fl), respectively, compared to A. Moreover, the values of MCH in groups D (15.14 ± 0.55 pg) and E (15.10 ± 0.36 pg) were 4.5% and 4.8% significantly (*p* < 0.05) lower, respectively, compared to A. The haemoglobin concentrations were 3.9%, 7.4%, 12% and 6.5% lower (*p* > 0.05; F = 2.692) in groups B (169.50 ± 3.75 g/L), C (163.40 ± 2.18 g/L), D (155.20 ± 4.66 g/L) and E (165.00 ±4.54 g/L), respectively, compared to A (176.40 ± 8.35 g/L) (see Table 8). Besides, there was 59.8% significant (*p* < 0.05; F = 4.014) increase in total leukocyte counts in group C (11.86 ± 1.00 × 10^9^/L) compared to A (7.42 ± 0.95 × 10^9^/L). The increased leukocyte counts were accompanied by 102.8% significant (*p* < 0.05; F = 2.961) increase in neutrophils in group C (3.61 ± 0.88 × 10^9^/L) compared to A (1.78 ± 0.35 × 10^9^/L), as well as 55.3% increase (*p* > 0.05; F = 2.677) in monocytes in group C (0.73 ± 0.11 ×10^9^/L) compared to A (0.47 ± 0.09 × 10^9^/L) (Table 8).

#### 2.3.4. Plasma Biochemical Parameters

The effects of repeated oral administration of different doses of CELE daily for 28 days on the plasma biochemical parameters of female ICR mice is presented on Table 9. There were statistically significant (*p* < 0.05) differences between groups in the plasma biochemical parameters of the mice as determined by one-way ANOVA (Table 9). The Tukey post hoc test showed that the mice treated with CELE had 77.7% significant (*p* < 0.05; F = 4.118) increase in creatinine level in group E (33.40 ± 2.93 µmol/L) compared to A (18.80 ± 1.62 µmol/L). However, the urea levels were 10.03% and 27.4% lower in groups C (10.31 ± 1.24 mmol/L; *p* > 0.05; F = 1.113) and E (8.45 ± 0.60 mmol/L; *p* < 0.05), respectively, compared to group A (11.64 ± 0.94 mmol/L). Furthermore, there was also 105.7% significant (*p* < 0.05; F = 4.063) increase in the plasma level of ALT in group D (265.80 ± 45.03 U/L) compared to A (129.20 ± 15.81 U/L). The plasma levels of AST were 13.1% and 4.8% higher (*p* > 0.05; F = 0.978) in groups C (421.87 ± 11.07 U/L) and E (390.90 ± 29.34 U/L), respectively, compared to group A (373.00 ± 8.93 U/L) (Table 9). In addition, the plasma levels of total proteins were 5.9% and 3.83% higher (*p* > 0.05; F = 0.625) in groups D (67.98 ± 4.32 g/L) and E (66.68 ± 1.51 g/L), respectively, compared to group A (64.22 ± 1.78 g/L). The plasma albumin was 9.70% lower (*p* > 0.05; F = 0.457) in group E (29.13 ± 4.05 g/L), compared to A (32.26 ± 1.25 g/L). However, there were 15.3% and 17.5% increases (*p* > 0.05; F = 0.927) in the plasma levels of globulins in groups D (36.84 ± 3.74 g/L) and E (37.55 ± 5.17 g/L), respectively, compared to group A (31.96 ± 2.06 g/L) (Table 9).

#### 2.3.5. Liver Lesion Scoring

**Figure 12:**
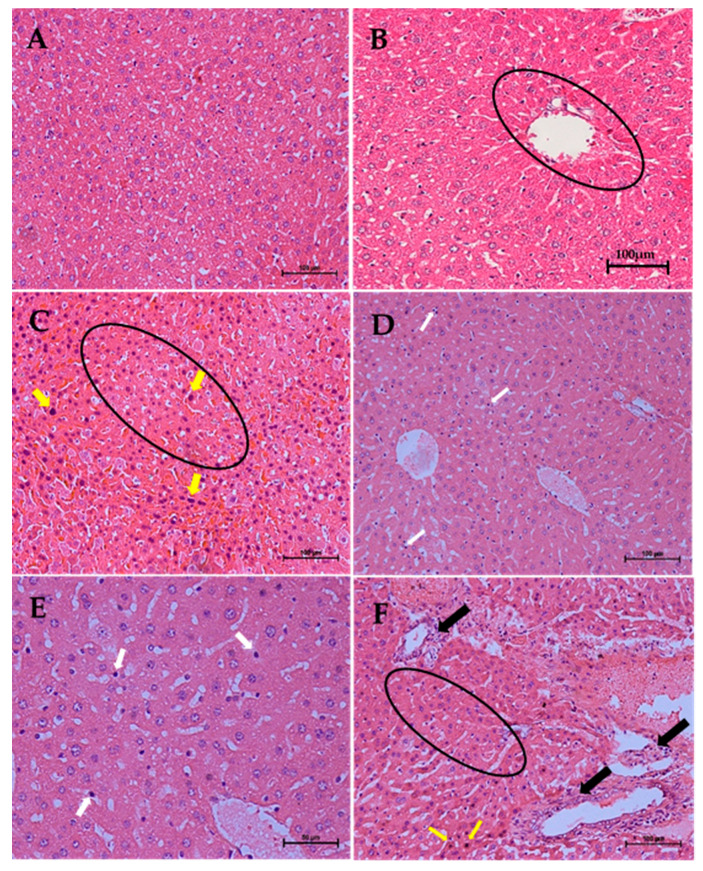
Effects of repeated oral administration of CELE for 28 days on the histology of liver of female ICR mice. Key: (**A**) Photomicrograph of a liver section (H&E stain ×200) from a mouse in group A (control) showing normal architecture of liver. (**B**) Photomicrograph of a liver section (H and E stain ×200) from a mouse in group A showing portal triad (encircled). (**C**) Photomicrograph of a liver section (H&E stain ×200) from a mouse in group E (1000 mg/kg CELE) showing eosinophilic cytoplasm (encircled) and pyknotic nuclei (yellow arrows) of the hepatocytes. (**D**) Photomicrograph of a liver section (H&E ×200) from a mouse in group E (1000 mg/kg) showing activated Kupffer cells (white arrows). (**E**) Higher magnification (H and E stain ×400) of D showing activation of Kupffer cells (white arrows). (**F**) Photomicrograph of a liver section (H and E stain ×200) from a mouse in group E (1000 mg/kg CELE), showing hepatitis (black arrows), eosinophilic cytoplasm (encircled) and pyknosis (yellow arrows) of the hepatocytes, H and E = haematoxylin and eosin, CELE = *Clinacanthus nutans* ethanolic leaf extract. Scale bars represent 100 µm.

The effects of repeated oral administration of different doses of CELE daily for 28 days on the histology of liver of female ICR mice is presented on Table 10. Kruskal-Wallis H test showed statistically significant (*p* < 0.05) differences in the lesion score between the different treatment groups (Table 10). Pairwise comparisons test revealed a mild activated Kupffer cells (Figure 12D,E), moderate sinusoidal dilatation and eosinophilic cytoplasm (Figure 12C) as well as mild pyknosis of the hepatocytes (Figure 12C,F). These were significantly (*p* < 0.05) higher in group E compared to A (Table 10). These findings corroborate those of plasma biochemical parameters, where there were statistically significant (*p* < 0.05) differences in the liver parameters between the control and treatment groups (Table 9).

#### 2.3.6. Kidney Lesion Scoring

**Figure 13:**
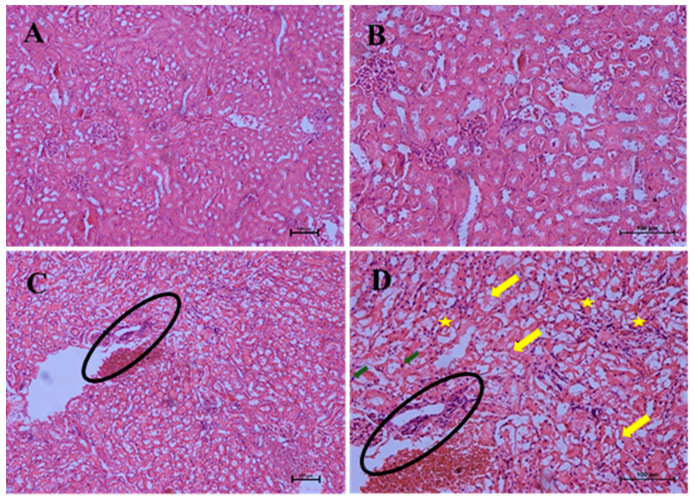
Effects of repeated oral administration of CELE for 28 days on the histology of kidney of female ICR mice. Key: (**A**) Photomicrograph of a kidney section (H and E stain ×100) from a mouse in group A showing normal architecture of kidney, (**B**) Higher magnification of A (×200), (**C**) Photomicrograph of a kidney section (H and E stain ×100) from a mouse in group E (1000 mg/kg CELE) showing cellular infiltrations (encircled) in the kidney. (**D**) Photomicrograph of a kidney section (H and E stain ×200) from a mouse in group E (1000 mg/kg CELE) showing cellular infiltrations in the kidney (encircled), protein casts (yellow arrows), eosinophilic cytoplasm (star) and pyknosis of the renal tubules (green arrow), CELE = *Clinacanthus nutans* ethanolic leaf extract, H and E = haematoxylin and eosin. Scale bars represent 100 µm.

The effects of repeated oral administration of different doses of CELE daily for 28 days on the histology of kidneys of female ICR mice are presented on Table 11. Kruskal–Wallis H test showed statistically significant (*p* < 0.05) differences in the lesion score between the different treatment groups (Table 11). Pairwise comparisons test revealed a moderate to severe renal tubular necrosis characterised by eosinophilic cytoplasm and pyknosis of the renal tubular cells (Figure 13D) in group E (2.10 ± 0.29) compared A (0.20 ± 0.20). Furthermore, there was significant (*p* < 0.05) mild nephritis (Figure 13C,D) in group E (1.30 ± 0.34) compared to A (0.00 ± 0.00). (Table 11).

## 3. Discussion

Analysis of bioactive compounds from a plant (also known as metabolomics) is considered a powerful discipline in plant sciences that is applicable in many aspects of plant biology, including aspects of growth and development, responses to external stimuli, genetics as well as nutritional requirements [22,23]. Phytochemical compounds are biologically important chemicals that occur naturally in plants. They may be responsible for colour and other organoleptic properties of plants. Phenolics, alkaloids, saponins, glycosides, terpenes, tannins, anthraquinones and steroids are some of the groups of phytochemical compounds identified in most medicinal plants [24,25]. These phytochemicals have been reported to be responsible for biological activities, including antioxidant, tissue protective, analgesic, antiulcer, antihypertensive, radioprotective and immunomodulatory effects, associated with most of the medicinal plants including *C. nutans* in both humans and animal studies [26]. Previous phytochemical investigation of *C. nutans* extracts revealed the presence of various bioactive compounds including C-glycosyl flavones [27], phytosterols, triterpenoid [28], stigmasterol, glycoglycerolipids [29], lupeol, b-sitosterol, belutin, sulphur-containing glycosides and some compounds related to chlorophyll a and chlorophyll b. The extraction method has a significant role on the phytochemical yield of *C. nutans* extract [30]. Alam et al. [6] reported that vitexin, iso-vitexin, schaftoside, isomollupentin 7-*O*-bglucopyranoside, orientin and iso-orientin were isolated from the *n*-butanol and water-soluble fractions of methanolic extract of this plant in Thailand.

The results of the LC/MS/MS analyses of the *C. nutans* ethanolic leaf extract in this study demonstrated the presence of myricetin, orientin, iso-orientin, vitexin, iso-vitexin, isookanin, apigenin and ferulic acid as some of the important bioactive compounds of the plant. However, three of these compounds—myricetin, isookanin and ferulic acid—were additional compounds identified in the *C. nutans* ethanolic leaf extract cultivated in Pahang-Malaysia, which were not among the compounds identified earlier by Huang et al. [17] and Chelyn et al. [18], as well as other recent literatures as reported by Khoo et al. [31].

The identification of orientin, isoorientin, vitexin and isovitexin in this study corroborates the earlier report by Huang et al. [17] where a total of seven compounds including shaftoside (apigenin-6-*C*-β-d-glucopyranosyl-8-*C*-α-l-arabinopyranoside), apigenin 6,8-*C*-α-l-pyranarabinoside, orientin, isoorientin, isovitexin and vitexin were identified from 30% ethanol extract of *C. nutans*. Similarly, Chelyn et al. [18] reported that shaftoside, iso-orientin, orientin, iso-vitexin and vitexin were the major flavonoids found in the leaves of *C. nutans* cultivated in Perak, Johor, and Negri Sembilan, Malaysia [18]. 

Vitexin and isovitexin are bioactive compounds found in many traditional Chinese medicine and in various medicinal plants [32]. Vitexin, which is otherwise known as apigenin-8-*C*-glucoside, has nowadays gained increasing attention because of its diverse pharmacological activities, including antioxidant, anticancer, anti-inflammatory, neuroprotective, etc. Isovitexin, on the other hand, is an isomer of vitexin also known as apigenin-6-*C*-glucoside, and it is also associated with wide range of biological activities [32].

Orientin is a water-soluble flavonoid *C*-glycoside that is chemically known as 2-(3,4-dihydroxyphenyl)-5,7-dihydrox-y-8[(2*S*,3*R*,5*S*,6*R*)-3,4,5-trihydroxy-6-(hydroxymethyl)]chromane-4-one. The compound has been isolated from different medicinal plants including *Ocimum sanctum*, *Phyllostachys* species (bamboo leaves), *Passiflora* species (passion flowers), *Trollius* species (Golden Queen) and *Jatropha gossypifolia* (Bellyache Bush) [33]. Orientin has been reported to be associated with various pharmacological activities including antibacterial, antiviral, anti-inflammatory, antioxidant and antiageing, among others [33]. Moreover, isoorientin is a flavone that is a 6-*C*-glucoside of luteolin. It has been reported to have antidiabetic, anti-inflammatory, proapoptotic and antioxidant activities.

The three additional compounds identified in this study were also reported to have various pharmacological activities. Myricetin is one of the common plant derived flavonoids that is well known for its nutraceutical activities [34]. It is one of the naturally occurring phenolic compounds in fruits, berries, vegetables, teas, medicinal plants and wines produced from various plants [34,35]. Myricetin was first isolated in India from the bark of *Myrica nagi* Thunb. (Myricaceae) as light yellow-coloured crystals [36]. The compound is recognised mainly for its iron-chelating, anticancer, antioxidant, anti-inflammatory and antidiabetic activities among others [34,37].

Ferulic acid belongs to the phenolic acids group that are commonly found in plant tissues [38]. It is mostly found in whole grains, grapes, parsley, spinach, oats, rhubarb and barley [39]. Similarly, ferulic acid was reported to have several physiological functions ranging from antioxidant, anti-inflammatory, antimicrobial, antidiabetic and anticancer activities [39]. It is also widely used in skin care formulations as a photoprotective agent and as an anti-skin photoageing process. However, ferulic acid is reported to have a high tendency for rapid oxidation [39,40,41,42], which could make it prone to toxicity and oxidative stress.

The presence of these compounds could be responsible for most of the pharmacological activities associated with *C. nutans*, including antitumor, anti-snake and insect bites, anti-VZV lesions and anti-hepatitis activities reported in the literature [17,43,44]. Although these compounds are reported to have potential pharmacological activities, it should be noted that the compounds could also have some adverse effects on normal cells, especially when consumed in large quantities.

The assessment of the toxic effect of plant is very necessary in evaluating its safety for both human and animal use. Evaluation of acute toxicity study of a plant or substance may perhaps provide significant information for identification of the targeted organs of the test substances following acute exposure [45]. The acute toxicity study of CELE in this study revealed that the extract at 2000 mg/kg did not cause any mortality or any signs of acute toxicity in the treated mice. The appetite and activities of the mice in both the two experimental groups were not affected by the administration of the extract throughout the period of observation. This report corroborates with the earlier reports by P’ng et al. [14], who reported that administration of *C. nutans* methanolic leaf extract at 900 and 1800 mg/kg is not associated with either mortality or any signs of acute toxicity in the treated mice. Similarly, Sajjaratul et al. [7] and Khoo et al. [12], respectively, reported that administration of 2000 mg/kg ethanolic extract and 5000 mg/kg aqueous extract of *C. nutans* did not cause any toxicity nor mortality in Sprague Dawley rats throughout the 14-day period of observation. 

The significant fluctuations in the body weight gain of the mice treated with 2000 mg/kg CELE once in this study could suggests that the extract could have affected the appetite of the mice at the beginning, but shortly after, the animals have adjusted, and the extract resulted in significant weight gain at the end of the experimental period. This is in agreement with P’ng et al. [14] and Khoo et al. [12], who also recorded a significant increase in the body weight of mice and rat, respectively, administered with methanolic and aqueous *C. nutans* extracts. Furthermore, the changes in the body weight gain pattern were similar to that reported by Khoo et al. [12], who observed significant alteration in carbohydrate metabolism, energy metabolism and amino acid metabolism in Sprague Dawley rats treated with 5000 mg/kg *C. nutans* aqueous extract 2 h post administration. Nonetheless, the metabolic expression collected 24 h, 5, 10 and 15 days post-administration showed that the rats have overcome the effects of the extracts and did not show accumulation of any toxicity biomarkers [12]. 

The oral administration of a high dose of the extract (CELE) at 2000 mg/kg once did not affect the haematological parameters of the ICR mice significantly in this study. This may suggest further that the extract at 2000 mg/kg may not have significant effects on the metabolism [46,47] of the treated mice. Correspondingly, Khoo et al. [12] reported that administration of *C. nutans* aqueous extract even at high dose of 5000 mg/kg did not affect the blood profile of Sprague Dawley rats following 14 days of observation.

Conversely, administration of the extract at 2000 mg/kg resulted in a significant increase in the levels of ALT, AST and CK, which may suggest that the extract has some adverse effects on the liver [48,49] of the treated mice. Correspondingly, histopathological evaluation of liver and kidneys suggested that the extract at this high dose of 2000 mg/kg might have induced mild histopathological lesions in the liver and kidneys of the treated mice. The histopathological lesions observed in this study, including cytoplasmic vacuolation and eosinophilic cytoplasm, although not significant, may suggest that the plant extract at 2000 mg/kg exhibited some degree of degenerative and necrotic effects [50,51] on the liver and kidney of the treated mice. The significant differences in the liver injury markers observed in this study contradicts the earlier reports by Khoo et al. [12], where administration of 5000 mg/kg aqueous extract of *C. nutans* did not cause any significant changes in the levels of AST, ALT, ALP and total bilirubin between the treated and untreated groups of rats; this may perhaps be due to the differences in the extraction solvents, as water has less ability to extracts phytochemicals compared to ethanol [30,52,53].

The subacute toxicity study of this research revealed significant alterations in certain parameters of the treated mice. The significant decrease in weight gain observed in the groups of mice treated with 500 mg/kg and 1000 mg/kg daily for 28 days could suggest that the extract affected the mice’s feed intake [54] or has resulted in the reduction in the deposition of fats [46,55]. Reduction in the body weight gain has been reported to be associated with toxicity following exposure to potential toxic chemical or substances in animals [56,57]. These findings are in agreement with the report of Chavalittumrong et al. [58], where a significant decrease body weight of male rats treated with 1.0 g/kg of *C. nutans* ethanolic extract daily for 90 days compared to the control group was reported. However, it is contrary to the report of Zakaria et al. [9], who reported that repeated oral administration of methanolic extract of *C. nutans* daily for 28 days did not affect the body weight of the mice even at the highest dose of 2500 mg/kg. This perhaps may be due to shorter duration of exposure [56,57] compared to the present study.

The repeated oral administration of the extract at 125 mg/kg, 250 mg/kg, 500 mg/kg and 1000 mg/kg daily for 28 days in this study did not affect the haematological parameters of the ICR mice significantly, suggesting that the metabolic processes of the mice were not significantly affected [59,60] by the administration of the extract.

The mean corpuscular volume (MCV) is used to classify anaemia as microcytic (below the normal range), normocytic (within the normal range) or macrocytic (above the normal range) [61,62]. Therefore, the significant alterations in the values of MCV observed in this study could suggest that the extract has macrocytic effect on the RBC of the treated mice. Similarly, the mean corpuscular haemoglobin (MCH) and the mean corpuscular haemoglobin concentration (MCHC) are also used as indices for diagnosis of anaemia. Decreased level of MCHC may indicate hypochromasia in early iron deficiency anaemia [63]. Therefore, the significant increase in the level of MCH observed in this study could suggests that the extract might have certain compounds capable of promoting haemoglobin production and this is in agreement with the report of Archibong et al. [61]. 

The results of plasma biochemical parameters showed that daily oral administration of CELE for 28 days at 500 and 1000 mg/kg doses has effect on some plasma biochemical parameters. The significant increase in creatinine level detected in the group of mice treated with 1000 mg/kg daily for 28 days could suggest that the extract especially at higher doses of 500 and 1000 mg/kg may possess some adverse effects on the kidneys of the ICR mice [48,64,65]. This is because creatinine, which is the product of creatine metabolism, is solely excreted by the kidneys; therefore, any injury to the kidneys may result in hypercreatinaemia [66]. These findings were similar to the report of Zakaria et al. [9], where there was significant increase in the level of creatinine in both male and female mice treated with both 500 and 1000 mg/kg *C. nutans* repeatedly for 28 days. However, Chavalittumrong et al. [58] reported a significant decrease in creatinine levels in the male rats treated with 1000 mg/kg *C. nutans* ethanolic extract daily for 90 days compared to the control. The significant increase in the levels of ALT observed in this study at 500 and 1000 mg/kg, could suggest that the extract at this high doses might have affected the normal function of liver in the treated mice [48,49,67]. This was further supported by the results of the histopathological evaluation of liver and kidney in this study.

The histopathological evaluation of liver and kidney of the experimental mice in this study revealed that daily oral administration of CELE for 28 days at 1000 mg/kg dose resulted in various hepatic and renal lesions. The significant hepatic degeneration and necrosis observed histopathologically in the group of mice administered with daily oral doses of CELE at 1000 mg/kg for 28 days were suggestive of toxic or adverse effects [7,50,51,68,69,70] of the extract on the animals, as earlier observed in the results of plasma biochemical parameters for hepatic injury markers (ALT and AST). Similarly, the renal tubular degeneration and necrosis observed in this research further supported the results of plasma biochemical parameters, providing further indication that administration of the extract at 1000 mg/kg is not safe [50,51] for the female ICR mice. Severe hepatic damage has been reported to be associated with oxidative stress and depletion of ATP leading to necrosis of the hepatocytes [71,72].

P’ng et al. [13] reported that Food and Agricultural Materials Inspection Centre (FAMIC) uses the acceptable daily intake (ADI) to determine the non-toxic level of a test substance to humans based on the non-observable adverse effect level (NOAEL) value obtained from animal trials [13]. Based on the results of this study, the NOAEL of ethanolic leaf extract of *C. nutans* was 250 mg/kg in mice, whereas the low observable adverse effect level was 500 mg/kg in mice. Therefore, the ADI of *C. nutans* ethanolic leaf extract was determined to be 2.5 mg/kg, according to the method described by P’ng et al. [13]

## 4. Materials and Methods 

### 4.1. Plant Materials

Fresh leaves of the plant *C. nutans* were obtained from Malaysian Agriculture Research and Development Institute (MARDI) research station, Muadzam Shah, Pahang-Malaysia, from July to October 2017. The leaves were washed to remove dirt and soil deposits, dried under the sun for 48 h to remove moisture content. Subsequently, the leaves were ground into powder form and stored at 4 °C for further analyses [73].

#### 4.1.1. Botanical Identification

The plant *C. nutans* was identified botanically at the Faculty of Science and Technology (FST), University Kebangsaan Malaysia (UKM). Its kingdom, family and species were confirmed. The voucher specimen for the plant was deposited in the herbarium of FST, UKM (UKMB40367).

#### 4.1.2. Extraction Procedure

The dried powdered leaves of *C. nutans* were extracted using absolute ethanol at the ration of 1:40. One hundred grams (100 g) of *C. nutans* powdered leaves was dissolved in 4000 mL of absolute ethanol in a clean glass flask [74]. The flasks was covered with aluminium foil and placed in an orbital shaker (Heidolph Unimax 1010, Schwabach, Germany) at 200 rpm for about 2 h at room temperature [7,75,76]. The extract was filtered twice using Whatman No. 1 filter paper and concentrated using rotary evaporator (BUCHI Rotavapor R-200, Flawil, Switzerland). The concentrated extracts were combined together [77]. The concentrated extracts were then freeze-dried in a freeze-dryer (The Virtis Company, Gardiner, NY, USA) and obtained in both freeze-dried (for LC-MS) and semisolid (for toxicity studies) forms and were stored at −20 °C and 4 °C respectively until needed for the experiments [78]. 

#### 4.1.3. Preparation of Extracts

Dry extract of the plant leaf was weighed and dissolved in the solvents (5% DMSO) to prepare the desired stock solution of the extracts from which the dose was calculated according to the mice’s body weight [79].

### 4.2. Liquid Chromatography Mass Spectrometry (LC-MS)

#### 4.2.1. Sample Preparation

One gram (1 g) of CELE was weighed into 1 mL of LC-MS-grade methanol (as solvent), the mixture was sonicated using Bransonic Ultra sonic cleaner (2510E-DTH, Branson Ultrasonics Corporation, Danbury, CT, USA) at 25 °C for 5 min to dissolve the solute appropriately. The samples were then filtered using satorius NY 0.45 µm filter paper into LC-MS (HPLC) vials. The vials were then taken into the LC-MS machine for analyses.

#### 4.2.2. Chemicals and Equipment

Standard compounds: LC-MS-grade acetonitrile (ACN), formic acid and methanol (MeOH) were obtained from Fisher Scientific (Fair Lawn, NJ, USA). LC-MS-grade water (18 MX) was prepared using a Millipore Milli-Q purification system (Millipore Corporation, Bedford, MA, USA). 

High-performance liquid chromatography (HPLC) separation was performed using a Thermo Scientific^TM^ Dionex Ultimate 3000 LC system (Thermo Fisher Scientific, Waltham, MA, USA) with a Thermo Hypersil Gold aQ (1.9 µm, 100 mm × 2.1 diameter). A Thermo Scientific Q Exactive Focus (Thermo Fisher Scientific, USA) equipped with a pump: HGP-3200RS, Autosampler: WPS3000TRS, column compartment: TCC3000RS, a degasser: SRD3400, Diode Array Detector (DAD), Orbitrap mass analyser with a heated-electrospray ionization (H-ESI II), and software of Xcalibur and Chromaleon was used for LC-MS and LC-MS/MS detection.

#### 4.2.3. Compound Identification

The method of Huang et al. [17] was employed with modifications for the qualitative identification of compounds in the crude extract of CELE. The high-performance liquid chromatography (HPLC) separation was performed with mobile phase consisting of two different solvents: C and D in linear gradient. Solvent C was 0.1% formic acid (*v*/*v*) in water whereas solvent D was methanol. The gradient was set at 1–90% D in C over a period of 45 min at a flow rate of 0.2 mL/min. The injection volume was 10 µL and the UV detector was set at 280 nm [17]. The eluent was monitored by a Thermo Scientific ion max API source (H-ESI II), under both positive and negative ion modes and scanned from *m*/*z* 190 to 800. ESI was conducted by using a needle voltage of 4.2 kV and 3.5 kV for positive and negative modes respectively under optimum collision energy level of 30. High-purity nitrogen (99.999%) was used as dry gas and at a flow rate of 12 L/min and capillary temperature at 320 °C. Nitrogen was used as nebulizer at 40 psi [80].

### 4.3. Acute and Subacute Toxicity Studies of C. nutans Ethanolic Leaf Extract

The acute and subacute toxicity effects of the plant were studied according to OECD guidelines 425 and 407, respectively; with slight modification on the selection of gender of the experimental animals for the subacute toxicity testing. We chose to test the toxic effects of *C. nutans* on female ICR mice only (but not on the male). This is because female animals were reported to be more susceptible to toxic substances than their male counterparts [81]. Moreover, OECD guideline 425 reported that “testing in one sex (usually females) is generally considered sufficient”. This would minimize the number of animals required to estimate the possible toxicity of the plant extract (*C. nutans*).

The experiment was conducted at the Animal Metabolism, Toxicology and Reproductive Centre (AMTREC), Malaysian Agricultural Research and Development Institute (MARDI), Serdang. This research adhered to the guide for the care and use of laboratory animals and was approved by the Animal Ethics Committee (AEC) of MARDI with the approval reference number: 20170717/R/MAEC00023. The mice were acclimatized for 1 week to the housing conditions, with temperature within the range of 22 to 25 °C, humidity at the range of 40% to 70% and balance of 12 h light/12 h dark cycle. The bedding and water were replaced regularly, and the cages were cleaned accordingly. Each mouse was placed in a polycarbonate plastic cage. The animal used in this study were purchased from a commercial vendor at Selangor, Malaysia.

A total of 10 8-week-old female mice were used for the acute toxicity study. The mice were divided into two groups of five mice each: groups A and B. Group A received distilled water and served as control group, while group B received a single dose of 2000 mg/kg CELE by oral gavage [82] using stainless steel needle. The extract and distilled water were administered at the volume of 1 mL per 100 g body weight of the average body weight of each group of mice. The mice were fasted for 2–3 h prior to the administration of the extracts. Thereafter, all mice had free access to water and commercial chow ad libitum throughout the period of observation. The mice were monitored for mortality, behavioural signs (restlessness, dullness, agitation) and sign of toxicity, daily for 14 days [7].

For the subacute toxicity study, a total of 25 8-week-old female mice were divided into 5 groups of 5 mice each: groups A (control), B (125 mg/kg), C (250 mg/kg), D (500 mg/kg) and E (1000 mg/kg). The extract (CELE) was dissolved in 5% dimethyl sulfoxide (DMSO) and administered to groups B, C, D and E accordingly. The extract was administered once daily for 28 days at a volume of 1 mL/100 g body weight, via oral gavage using stainless steel needle. Group A received distilled water (vehicle) only at the same 1 mL/100 g body weight. The mice were monitored daily for any signs of toxicity and sacrificed on day 29 of the experiment using CO_2_ chamber.

#### 4.3.1. Determination of Weekly Body Weight and Body Weight Gain

The body weight of each mouse in each of the experimental groups was measured weekly using electric weighing scale and recorded according to the method of Sajjaratul et al. [7]. The weekly body weight gain was calculated by subtracting the previous weekly body weight of each mouse from that the current week.

#### 4.3.2. Collection of Blood and Organ Samples

The mice were sacrificed humanely on days 15 and 29 of the experiments for the acute and subacute toxicity studies respectively, using CO_2_ chamber [83,84]. Blood samples were collected from the heart [83] into clean bottles containing EDTA as an anticoagulant for haematological and blood chemistry analysis [82,84,85]. All mice were subjected to gross necropsy and tissues were collected from liver, kidney, heart, brain, spleen, lungs and uterus. The weight of each organ was measured and recorded. The relative organs weight was calculated by dividing the weight of each organ with the body weight of the corresponding mouse in each group [79].

$$Relative\;organ\;weight=\frac{organ\;weight}{body\;weight}\times 100$$

#### 4.3.3. Haematological Analyses

The blood samples collected were analysed for complete blood count using an automated haematology analyser (ABC Vet^®^, ABX Diagnostics, Montpellier, France) for the total RBC, WBC, platelet count, haemoglobin (Hb) concentration, mean corpuscular volume (MCV) and mean corpuscular haemoglobin concentration (MCHC). Blood smears were prepared and stained with Wright stain and examined under a light microscope. Differential WBC count was determined manually by counting 100 WBC on the blood smears. The absolute values of each type of WBC (neutrophils, eosinophils, basophils, lymphocytes and monocytes) was calculated by multiplying the percentage of each WBC type to the total WBC count from the automated analyser [7,86].

#### 4.3.4. Clinical Biochemistry Analysis

The blood samples collected in EDTA bottles were centrifuged for 15 min at 3000 rpm to obtain plasma for biochemical analyses [82,84,85,87,88,89,90]. The plasma was further analysed for urea, creatinine, creatinine kinase (CK), total protein (TP), albumin (ALB), aspartate aminotransferase (AST) and alanine aminotransferase (ALT) using a fully automated clinical chemistry analyser (BioLis 24i Chemistry Analyzer, Tokyo, Japan) [7,84,86]. The values for globulins were calculated manually by subtracting the values of albumin from that of total protein [84].

#### 4.3.5. Histopathological Evaluation

Post-mortem was conducted on each of the mouse, liver and kidneys were collected for histopathological evaluation at the histopathology laboratory, Faculty of Veterinary Medicine, Universiti Putra Malaysia. Collected organs were fixed in 10% neutral buffered formalin and processed further for haematoxylin and eosin stain as described by Nurul et al. [91] and Aliyu et al. [84]. The severity of each of the lesions was also scored according to the method of Nurul et al. [91] with modifications. The scores of each lesion and its interpretation is presented on Table 12.

### 4.4. Statistical Analysis

Statistical analyses were performed using *IBM *SPSS statistics version 23 [84]. Data was presented as mean ± standard error of the mean (SEM). Results from the body weight, relative organ weights, haematological and plasma biochemical analyses were accordingly subjected to Student’s *t* test statistical tool (between two treatment groups) as well as one-way analysis of variance (ANOVA) statistical tool with Tukey post hoc test (between three or more treatment groups). However, the results from the histopathological lesion scoring were analysed using nonparametric Mann-Whitney U test tool (between two treatment groups) and nonparametric Kruskal-Wallis H statistical tool (between three or more treatment groups). *p*-values < 0.05 were considered statistically significant. 

## 5. Conclusions

In conclusion, *C. nutans* ethanolic leaf extract cultivated in Pahang-Malaysia contained additional bioactive compounds compared to those cultivated in Perak, Johor and Negri Sembilan as reported earlier in the literature. Moreover, the lethal dose 50 (LD_50_) of CELE in mice is greater than 2000 mg/kg, as there was no mortality observed. Moreover, single oral administration of the extracts at 2000 mg/kg has no toxic effects on the haematological parameters of ICR mice; however, administration of the extracts once at 2000 mg/kg induced mild hepatic and renal histological alterations in the mice. Similarly, repeated daily oral administration of CELE for 28 days induced mild to moderate hepatic degeneration at 500 mg/kg and hepatic and renal necrosis at 1000 mg/kg in female ICR mice. Therefore, the plant extract should be taken with caution as food supplement and/or alternative medicine. It is recommended that the subchronic and even chronic toxicity effects of the extract be evaluated.

## Figures and Tables

**Table 1:** Bioactive compounds detected in *Clinacanthus nutans* ethanolic leaf extract (CELE).

S/N	Rt (min)	Tentative Compounds	[M − H]^−^ (*m*/*z*)	Fragment Ions
1	3.47	Myricetin	317.0465	99, 112, 116, 136, 145, 152, 161, 180, 183, 198, 220, 228
2	13	Orientin	447.0912	269.1028, 285.0403, 299.0561, 311.0564, 327.0513
3	17.27	Iso orientin	447.0912	327, 357, 299, 285
4	17.45	Vitexin	431.0981	117.0331, 161.0232, 283.0612, 311.0563, 341.0667
5	18.48	Isovitexin	431.0981	283,311,269,323,341
6	27.44	Isookanin	287.1218	89, 93, 151, 154, 169, 170, 178
7	28.48	Apigenin	269.0455	151,159,117,107
8	30.93	Ferulic acid	193.0863	63, 79, 80, 89, 163, 177, 178

**Table 2:** Relative organ weights in % (mean ± SEM) of female ICR mice in acute toxicity study of CELE.

Organs	A	B
Liver	5.39 ± 0.44	5.70 ± 0.33
Right Kidney	0.71 ± 0.08	0.60 ± 0.04
Left Kidney	0.79 ± 0.08	0.81 ± 0.10
Spleen	0.59 ± 0.10	0.57 ± 0.05
Heart	0.65 ± 0.07	0.62 ± 0.10
Lungs	1.49 ± 0.17	1.07 ± 0.14
Brain	1.25 ± 0.08	1.21 ± 0.04
Uterus	1.95 ± 0.31	2.10 ± 0.30

Key: CELE = *Clinacanthus nutans* ethanolic leaf extract, A = Control, B = 2000 mg/kg CELE, the differences between groups A and B were not statistically significant (*p* > 0.05).

**Table 3:** Haematological parameters (mean ± SEM) of female ICR mice in acute toxicity study of CELE.

Parameters	A	B
Red Blood Cells (×10^12^/L)	11.00 ± 0.12	11.28 ± 0.48
Haemoglobin (g/L)	176.80 ± 2.87	181.80 ± 5.70
PCV (l/l)	0.38 ± 0.01	0.37 ± 0.02
Platelets (×10^9^/L)	1308.40 ± 32.66	1252.00 ± 204.80
MCV (fl)	62.40 ± 0.51	62.80 ± 0.97
MCH (pg)	16.10 ± 0.26	16.14 ± 0.37
MCHC(g/L)	257.80 ± 3.07	257.20 ± 3.89
Plasma Proteins (g/L)	61.60 ± 2.14	61.60 ± 3.54
White Blood Cells (×10^9^/L)	7.07 ± 1.06	7.16 ± 0.86
Neutrophils (×10^9^/L)	1.51 ± 0.46	1.57 ± 0.21
Lymphocytes (×10^9^/L)	5.08 ± 0.64	5.16 ± 0.60
Monocytes (×10^9^/L)	0.40 ± 0.07	0.43 ± 0.04
Eosinophils (×10^9^/L)	0.08 ± 0.06	0.00 ± 0.00
Basophils (×10^9^/L)	0.00 ± 0.00	0.00 ± 0.00

Key: CELE = *Clinacanthus nutans* ethanolic leaf extract, A = Control, B = 2000 mg/kg CELE, MCV = mean corpuscular volume, MCH = mean corpuscular haemoglobin, MCHC = mean corpuscular haemoglobin concentration.

**Table 4:** Biochemical parameters (mean ± SEM) of female ICR mice in acute toxicity study of CELE.

Parameters	A	B
Urea (mmol/L)	9.60 ± 0.32	9.24 ± 0.66
Creatinine (µmol/L)	35.20 ± 2.42	29.20 ± 1.50
ALT (U/L)	101.60 ± 5.75	289.60 ± 30.99*
AST (U/L)	171.00 ± 10.89	344.60 ± 45.92*
CK (U/L)	327.60 ± 35.71	549.20 ± 66.05*
Total Protein (g/L)	63.56 ± 1.71	59.86 ± 2.17
Albumin (g/L)	34.10 ± 0.73	32.00 ± 0.45
Globulins (g/L)	29.60 ± 1.21	28.00 ± 2.00

Key: CELE = *Clinacanthus nutans* ethanolic leaf extract, A = Control, B = 2000 mg/kg CELE, ALT = alanine aminotransferase, AST = aspartate aminotransferase, CK = creatinine kinase; Values in the same row with asterisk differ significantly (*p* < 0.05)**.**

**Table 5:** Liver lesion scores (mean ± SEM) for female ICR mice in acute toxicity study of CELE.

Lesions	A	B
Hydropic degeneration	0.50 ± 0.32	1.20 ± 0.37
Eosinophilic cytoplasm	0.20 ± 0.24	0.60 ± 0.20
Pyknosis	0.30 ± 0.30	0.50 ± 0.32
Karyolysis	0.00 ± 0.00	0.00 ± 0.00
Sinusoidal dilatation	0.40 ± 0.24	0.60 ± 0.24
Activated Kupffer cells	0.20 ± 0.20	0.20 ± 0.20
Inflammation	0.00 ± 0.00	0.00 ± 0.00
Regeneration	0.50 ± 0.32	0.70 ± 0.44

Key: CELE = *Clinacanthus nutans* ethanolic leaf extract, A = Control, B = 2000 mg/kg CELE.

**Table 6:** Kidney lesion scores (mean ± SEM) for female ICR mice in acute toxicity study of CELE.

Lesions	A	B
Hydropic degeneration	0.00 ± 0.00	0.00 ± 0.00
Eosinophilic cytoplasm	0.00 ± 0.00	0.20 ± 0.20
Pyknosis	0.00 ± 0.00	0.00 ± 0.00
Karyolysis	0.00 ± 0.00	0.00 ± 0.00
Nephritis	0.00 ± 0.00	0.20 ± 0.20
Protein casts	0.00 ± 0.00	0.00 ± 0.00
Cellular Casts	0.00 ± 0.00	0.00 ± 0.00
Granular Casts	0.00 ± 0.00	0.00 ± 0.00

Key: CELE = *Clinacanthus nutans* ethanolic leaf extract, A = Control, B = 2000 mg/kg CELE.

**Table 7:** Relative organ weights in % (mean ± SEM) of female ICR mice in subacute toxicity study of CELE.

Organs (%)	A	B	C	D	E
Liver	6.81 ± 0.85	5.55 ± 0.38	5.53 ± 0.34	6.17 ± 0.54	5.86 ± 0.41
Right Kidney	0.79 ± 0.11	0.76 ± 0.09	0.79 ± 0.05	0.81 ± 0.05	0.86 ± 0.13
Left Kidney	0.75 ± 0.13	0.78 ± 0.09	0.78 ± 0.04	0.89 ± 0.09	0.81 ± 0.10
Spleen	0.67 ± 0.20	0.40 ± 0.02	0.69 ± 0.13	0.79 ± 0.16	0.56 ± 0.04
Heart	0.59 ± 0.09	0.56 ± 0.03	0.55 ± 0.04	0.52 ± 0.05	0.56 ± 0.03
Lungs	1.22 ± 0.21	1.02 ± 0.10	1.11 ± 0.08	1.29 ± 0.19	1.30 ± 0.08
Brain	1.64 ± 0.17	1.48 ± 0.10	1.58 ± 0.03	1.61 ± 0.08	1.63 ± 0.10
Uterus	2.35 ± 0.25	1.96 ± 0.26*	1.46 ± 0.29*	1.43 ± 0.15*	1.99 ± 0.37*

Key: CELE = *Clinacanthus nutans* ethanolic leaf extract, A = control, B = 125 mg/kg CELE, C = 250 mg/kg CELE, D = 500 mg/kg CELE, E = 1000 mg/kg CELE, values in the same row with asterisk are significantly different at *p* < 0.05.

**Table 8:** Haematological parameters (mean ± SEM) of female ICR mice in subacute toxicity study of CELE.

Parameters	A	B	C	D	E
RBC (×10^12^/L)	11.15 ± 0.65	9.84 ± 0.38	10.29 ± 0.28	10.26 ± 0.18	10.99 ± 0.38
Hb (g/L)	176.40 ± 8.35	169.50 ± 3.75	163.40 ± 2.18	155.20 ± 4.66	165.00 ± 4.54
PCV (l/l)	0.48 ± 0.03	0.44 ± 0.02	0.39 ± 0.02	0.41 ± 0.03	0.43 ± 0.01
Platelets (×10^9^/L)	989.80 ± 197.4	891.50 ± 205.0	995.60 ± 224.3	1280.40 ± 126.	860.20 ± 195.2
MCV (fl)	65.80 ± 0.97	68.25 ± 1.80*	63.80 ± 1.36	62.20 ± 1.69	61.00 ± 1.48*
MCH (pg)	15.86 ± 0.28	17.33 ± 0.56*	15.92 ± 0.43	15.14 ± 0.55*	15.10 ± 0.36
MCHC (g/L)	241.80 ± 3.54	248.50 ± 4.03	248.80 ± 2.75	243.00 ± 4.32	255.20 ± 3.02
PP (g/L)	72.00 ± 2.00	73.00 ± 2.05	79.40 ± 6.66	77.40 ± 5.12	72.00 ± 1.67
WBC (×10^9^/L)	7.42 ± 0.95	9.18 ± 1.03	11.86 ± 1.00*	8.16 ± 0.50	9.47 ± 0.56
Neutrophils (×10^9^/L)	1.78 ± 0.35	1.60 ± 0.22	3.61 ± 0.88*	2.57 ± 0.25	2.85 ± 0.34
Lymphocytes (×10^9^/L)	5.14 ± 0.76	7.14 ± 0.81	7.48 ± 0.97	4.98 ± 0.76	6.41 ± 0.40
Monocytes (×10^9^/L)	0.47 ± 0.09	0.42 ± 0.05	0.73 ± 0.11	0.56 ± 0.04	0.51 ± 0.06
Eosinophils (×10^9^/L)	0.03 ± 0.03	0.02 ± 0.02	0.04 ± 0.04	0.05 ± 0.05	0.06 ± 0.04
Basophils (×10^9^/L)	0.00 ± 0.00	0.00 ± 0.00	0.00 ± 0.00	0.00 ± 0.00	0.00 ± 0.00

Key: CELE = *Clinacanthus nutans* ethanolic leaf extract, A = control, B = 125 mg/kg CELE, C = 250 mg/kg CELE, D = 500 mg/kg CELE, E = 1000 mg/kg CELE, values in the same row with asterisk were significantly different from the control group (*p* < 0.05), RBC = red blood cells, Hb = haemoglobin, PCV = packed cell volume, PP = plasma protein, WBC = white blood cells, MCV = mean corpuscular volume, MCH = mean corpuscular haemoglobin, MCHC = mean corpuscular haemoglobin concentration.

**Table 9:** Biochemical parameters (mean ± SEM) of female ICR mice in subacute toxicity study of CELE.

Parameters	A	B	C	D	E
Urea (mmol/L)	11.64 ± 0.94	10.98 ± 1.20	10.31 ± 1.24	11.06 ± 1.61	8.45 ± 0.60*
Creatinine (µmol/L)	18.80 ± 1.62	19.40 ± 3.23	24.40 ± 3.54	24.80 ± 2.73	33.40 ± 2.93*
ALT (U/L)	129.20 ± 15.81	185.53 ± 24.26	209.53 ± 11.44	265.80 ± 45.0*	173.10 ± 10.38
AST (U/L)	373.00 ± 8.93	362.67 ± 34.42	421.87 ± 11.07	362.60 ± 30.78	390.90 ± 29.34
CK (U/L)	627.20 ± 20.53	647.20 ± 95.80	634.27 ± 32.34	625.20 ± 99.88	592.80 ± 121.3
Total Protein (g/L)	64.22 ± 1.78	63.83 ± 1.91	63.87 ± 1.10	67.98 ± 4.32	66.68 ± 1.51
Albumin (g/L)	32.26 ± 1.25	32.56 ± 0.94	31.52 ± 0.65	31.14 ± 0.81	29.13 ± 4.05
Globulins (g/L)	31.96 ± 2.06	31.27 ± 1.15	32.11 ± 1.43	36.84 ± 3.74	37.55 ± 5.17

Key: CELE = *Clinacanthus nutans* ethanolic leaf extract, A = control, B = 125 mg/kg CELE, C = 250 mg/kg CELE, D = 500 mg/kg CELE, E = 1000 mg/kg CELE, ALT = alanine aminotransferase, AST = aspartate aminotransferase, CK = creatinine kinase, values in the same row with asterisk differ significantly (*p* < 0.05).

**Table 10:** Liver lesion scores (mean ± SEM) for female ICR mice in subacute toxicity study of CELE.

Lesions	A	B	C	D	E
Hydropic Degeneration	0.30 ± 0.30	0.60 ± 0.24	0.80 ± 0.34	0.80 ± 0.37	0.90 ± 0.56
Eosinophilic Cytoplasm	0.00 ± 0.00	0.40 ± 0.24	0.50 ± 0.32	1.00 ± 0.42	2.00 ± 0.22*
Pyknosis	0.00 ± 0.00	0.30 ± 0.30	0.30 ± 0.30	1.40 ± 0.40	1.90 ± 0.29*
Karyolysis	0.00 ± 0.00	0.00 ± 0.00	0.00 ± 0.00	0.00 ± 0.00	0.50 ± 0.50
Sinusoidal Dilatation	0.00 ± 0.00	1.10 ± 0.33	1.80 ± 0.20	2.30 ± 0.12*	2.30 ± 0.12*
Activated Kupffer Cells	0.00 ± 0.00	0.30 ± 0.30	1.10 ± 0.33	1.00 ± 0.47	1.70 ± 0.20*
Inflammation	0.00 ± 0.00	0.20 ± 0.20	0.60 ± 0.40	0.50 ± 0.32	0.40 ± 0.24
Regeneration	1.10 ± 0.46	0.50 ± 0.50	0.20 ± 0.20	0.80 ± 0.34	0.80 ± 0.34

Key: CELE = *Clinacanthus nutans* ethanolic leaf extract, A = control, B = 125 mg/kg CELE, C = 250 mg/kg CELE, D = 500 mg/kg CELE, E = 1000 mg/kg CELE, asterisk means statistical difference regarding group A (control).

**Table 11:** Kidney lesion scores (mean ± SEM) for female ICR mice in subacute toxicity study of CELE.

Lesions	A	B	C	D	E
Hydropic Degeneration	0.00 ± 0.00	0.00 ± 0.00	0.00 ± 0.00	0.20 ± 0.20	0.40 ± 0.24
Eosinophilic Cytoplasm	0.20 ± 0.20	0.20 ± 0.20	0.20 ± 0.20	1.40 ± 0.19	2.1 ± 0.29*
Pyknosis	0.00 ± 0.00	0.00 ± 0.00	0.00 ± 0.00	0.00 ± 0.00	0.90 ± 0.40
Karyolysis	0.00 ± 0.00	0.00 ± 0.00	0.00 ± 0.00	0.30 ± 0.30	1.20 ± 0.49
Nephritis	0.00 ± 0.00	0.00 ± 0.00	0.00 ± 0.00	0.90 ± 0.24	1.3 ± 0.34*
Protein Casts	0.20 ± 0.20	0.20 ± 0.20	0.30 ± 0.30	0.20 ± 0.20	0.80 ± 0.34
Cellular Casts	0.00 ± 0.00	0.00 ± 0.00	0.00 ± 0.00	0.00 ± 0.00	0.00 ± 0.00
Granular Casts	0.40 ± 0.24	0.20 ± 0.20	0.50 ± 0.32	0.00 ± 0.00	0.30 ± 0.30

Key: CELE = *Clinacanthus nutans* ethanolic leaf extract, A = control, B = 125 mg/kg CELE, C = 250 mg/kg CELE, D = 500 mg/kg CELE, E = 1000 mg/kg CELE, * = significantly different at *p* < 0.05.

**Table 12:** Interpretation of scores in liver and kidney lesion scoring for the toxicity studies of CELE in ICR mice.

* Score	Percentage	Severity
0	NONE	NONE
1	Less than 10%	Mild
1.5	10–30%	Mild-moderate
2	30–50%	Moderate
2.5	50–70%	Moderate-severe
3	More than 70%	Severe

Key: CELE = *Clinacanthus nutans* ethanolic leaf extract, * = Modified from Sajjaratul et al. [7] and Nurul et al. [91].
